# Impacts of short-term exposure to ambient air pollutants on outpatient visits for respiratory diseases in children: a time series study in Yichang, China

**DOI:** 10.1265/ehpm.24-00373

**Published:** 2025-03-12

**Authors:** Lu Chen, Zhongcheng Yang, Yingdong Chen, Wenhan Wang, Chen Shao, Lanfang Chen, Xiaoyan Ming, Qiuju Zhang

**Affiliations:** 1 Department of Biostatistics, School of Public Health, Harbin Medical University, Harbin, Heilongjiang, China; 2 Yichang Center for Disease Control and Prevention, Yichang, Hubei, China

**Keywords:** Air pollutants, Children’s respiratory diseases, DLNM model

## Abstract

**Background:**

There is growing evidence that the occurrence and severity of respiratory diseases in children are related to the concentration of air pollutants. Nonetheless, evidence regarding the association between short-term exposure to air pollution and outpatient visits for respiratory diseases in children remains limited. Outpatients cover a wide range of disease severity, including both severe and mild cases, some of which may need to be transferred to inpatient treatment. This study aimed to quantitatively evaluate the impact of short-term ambient air pollution exposure on outpatient visits for respiratory conditions in children.

**Methods:**

This study employed data of the Second People’s Hospital of Yichang from January 1, 2016 to December 31, 2023, to conduct a time series analysis. The DLNM approach was integrated with a generalized additive model to examine the daily outpatient visits of pediatric patients with respiratory illnesses in hospital, alongside air pollution data obtained from monitoring stations. Adjustments were made for long-term trends, meteorological variables, and other influencing factors.

**Results:**

A nonlinear association was identified between PM_2.5_, PM_10_, O_3_, NO_2_, SO_2_, CO levels and the daily outpatient visits for respiratory diseases among children. All six pollutants exhibit a hysteresis impact, with varying durations ranging from 4 to 6 days. The risks associated with air pollutants differ across various categories of children’s respiratory diseases; notably, O_3_ and CO do not show statistical significance concerning the risk of chronic respiratory conditions. Furthermore, the results of infectious respiratory diseases were similar with those of respiratory diseases.

**Conclusions:**

Our results indicated that short-term exposure to air pollutants may contribute to an increased incidence of outpatient visits for respiratory illnesses among children, and controlling air pollution is important to protect children’s health.

**Supplementary information:**

The online version contains supplementary material available at https://doi.org/10.1265/ehpm.24-00373.

## Background

Outdoor air pollution originates from geological and biological factors, and the majority one is anthropogenic [1]. The pollutants include particulate matter such as PM_2.5_ and PM_10_, and gaseous pollutants like O_3_, SO_2_, NO_2_, and CO, which are closely linked to industrial emissions, vehicular exhaust, and domestic pollution. Both short and long exposure can cause significant impacts on various organ systems within the human body [2, 3]. The respiratory system serves as a primary interface for gas exchange, due to its direct contact with air pollutants at high frequencies, it is particularly vulnerable to adverse impacts [4].

Children as a special group are more susceptible to air pollutants than adults [5]. To begin with, their respiratory systems are undergoing physiological development, which makes them relatively delicate and unstable. Lung maturation continues after birth through early childhood and may extend into adolescence, involving ongoing development of the airways and alveoli during this time [6, 7]. Lung function typically reaches its peak in early adulthood and subsequently declines with age [8]. Secondly, children are subjected to higher relative doses of air pollutants. The extent of exposure to inhaled pollutants is influenced by factors such as pollutant concentration, duration of exposure, and the volume of air inhaled. In children, nasal filtration is associated with more oral breaths, a smaller airway area, a higher rate of air change per body weight, and more time spent outdoors, all of which increase air pollution exposure [9–11]. In view of the distinctive characteristics of children’s physiological development and behavioral patterns, the impact of air pollutants on children’s respiratory diseases should be paid more attention.

Furthermore, risks associated with childhood cannot be extrapolated from adult data. Thus, research specifically focused on pediatric populations is essential to provide robust scientific evidence that can assist pediatricians, educators, parents, and other stakeholders in making informed decisions. For instance, a multi-city study conducted in China revealed that the influence of air pollutants on influenza, particularly nitrogen dioxide and sulfur dioxide, is more significant in children than in adults [12]. A study in southwestern China has demonstrated that the impact of air pollutants on respiratory diseases varies across different age groups [13]. For acute bronchitis, NO_2_ seriously harmed the elderly, while PM_10_, PM_2.5_ and SO_2_ had the greatest influence on people <20 years old [13]. A time-series analysis of respiratory diseases conducted in Lanzhou, China, suggested that children aged 0 to 14 years are more vulnerable to the adverse impacts of air pollution compared to other age groups [14]. Although the above-mentioned studies included children, they were not regarded as the main population for analyzing the relationship between pollutants and respiratory diseases. This study took children aged 0 to 14 as the research subjects, examined the relationship between six pollutants and the incidence of respiratory diseases, and focused on the subcategories of respiratory diseases. In addition, the sensitivity analysis and the subgroup analysis which were grouped according to gender and age were conducted. Providing evidence for the prevention and control of respiratory diseases in Yichang.

## Methods

### Introduction to Yichang

Yichang is located in 110°15′∼112°04′ east longitude and 29°56′∼31°34′ north latitude, a prefecture-level city in the southwestern of Hubei Province. It serves as a sub-central urban center within the province and plays a significant role in the urban agglomeration along the middle Yangtze River. According to the results of the seventh census, the permanent population of Yichang reached approximately 4.018 million, and children aged 0–14 accounted for 11.72% of the resident. The city lies within a transitional zone between the Middle Subtropical Zone and North Subtropical Zone, characterized by a subtropical monsoon humid climate with four distinct seasons. Yichang CDC has implemented environmental health impact assessments and protective measures, conducting long-term continuous monitoring of air pollution and climate change at designated sites to evaluate their impacts on population health.

### Data collection

The Second People’s Hospital of Yichang is a national third-class A hospital integrating medical treatment, teaching, scientific research and prevention, with over 650,000 outpatient visits annually. The outpatient records of the hospital from January 1, 2016 to December 31, 2023 were collected. These records included age, gender, outpatient diagnoses classified according to the International Classification of Diseases (ICD-10) and visit date. The inclusion criteria were as follows: (1) children aged 0–14 years old; (2) ICD codes were between J00–J99; (3) visit date from January 1, 2016 to December 31, 2023. Discharge criteria: repeat record. ICD code J00–J22, including acute upper respiratory infection, influenza and pneumonia, acute lower respiratory infection and other diseases were defined as infectious respiratory diseases (IRD). Meanwhile, ICD code J30–J47, including chronic rhinitis, bronchitis, asthma and other diseases were defined as chronic respiratory diseases (CRD). Ethical approval and individual consent are not required for this study since only aggregated non-identifiable data was employed.

Both the concentration of pollutants and meteorological indicators were obtained from the Yichang Municipal Environmental Protection Bureau from January 1, 2016 to December 31, 2023. The daily concentrations of five pollutants, PM_2.5_, PM_10_, SO_2_, NO_2_ and CO, as well as the 8-hour average concentration of ozone (O_3_), were continuously measured at five air quality monitoring stations in Yichang urban area. The average concentration for each across these five monitoring stations was calculated to represent the total daily exposure. Additionally, meteorological factors included daily average temperature (TEMP), average relative humidity (RH), and daily average wind speed (WS). To address missing values in daily average temperature data, the average value of maximum and minimum temperatures was recruited to fill it. Furthermore, multiple interpolation methods were employed to process daily average wind speed data in order to adjust for weather factors influencing outpatient treatment.

### Statistical methods

#### Part I Descriptive statistics

First of all, the original data were organized into time series corresponding in days. Our study presented a comprehensive analysis of the statistics concerning pollutants and meteorological factors, visualized the time series, and described the correlations between these variables. Considering the non-normal distribution characteristic of the data, median and interquartile range were employed as the primary descriptive statistics, while Spearman correlation analysis was employed to assess correlations. Meanwhile, we also calculated other statistics such as mean, standard deviation, etc. For the outpatient visits, we only used the daily number of visits for description.

#### Part II DLNM model

Considering nonlinear relationship between air pollutants and health impacts and the hysteresis impact of exposure, this study combined the distributed lag nonlinear model (DLNM) [15] with the generalized additive model. Considering the dispersed Poisson distribution of outpatient visits, Quasi-Poisson regression was used; air pollutants were incorporated into the cross-basis in a nonlinear format; 14 days was defined as lag period to account for both immediate and delayed impacts. The outcome of disease was divided into infectious respiratory diseases (IRD), and chronic respiratory diseases (CRD). Additionally, average daily temperature, relative humidity, and wind speed were included in the model to adjust for meteorological influences. Drawing from prior research findings, a natural spline function (ns) was applied with degrees of freedom set at 3 [16]. A time variable was introduced to control for long-term trend impacts and seasonal impacts using another natural spline function with degrees of freedom set at 8 per year. Weekday variable was also included in the basic model to adjust for the impacts of the day of the week. The structure of the model was as follows:

$$\begin{array}{rl} g(Y) & =\text{cb}(P_{i})+\text{ns}(\text{Time},8\text{df}/y)+\text{ns}(\text{TEMP},3\text{df})\\ & \;+\text{ns}(\text{RH},3\text{df})+\text{ns}(\text{WS},3\text{df})+\text{dow}+\alpha \end{array}$$

*g*( ) was the connection function, *Y* was the expected value of daily outpatient visits, *cb*(*Pi*) was the cross basis of each air pollutant, including PM_2.5_, PM_10_, SO_2_, NO_2_, CO, O_3_, *ns*( ) was the natural spline function, *df* was the degree of freedom, *Time* represented the time trend, *dow* was the day of the week. *TEMP* was the daily average temperature, *RH* was the average relative humidity, and *WS* was the wind speed.

#### Part III Selection of reference levels for exposure

We established single model for each pollutant. For the exposures were divided into four different level groups according the quartiles, and the lower quartile (*P*_25_) taken as reference to estimate the risk impact. The relative risk (*RR*) values for each lag day from lag0 to lag14 at *P*_75_ were calculated and identified the maximum lag day when RR value was not statistically significant. Then, the cumulative associations were estimated according the maximum lag day.

#### Part IV Subgroup analysis and sensitivity analysis

Participants were categorized into boys and girls based on gender, with age groups defined as 0–5 years for preschoolers and 6–14 years for school-aged children. The data are grouped by season, with April to October as the warm season and November to March as the cold season. Subgroup analyses were conducted to compare differences between these subgroups using Z-tests.

Given the potential collinearity of air pollutants affecting hospitalization risk, a dual pollution model was established to adjust for the impacts of other pollutants; six pollutants were combined in pairs within this model. The primary pollutant was incorporated into the model as a cross-basis, while the secondary pollutant was introduced using a natural spline function. Additionally, sensitivity analyses were performed by varying the degrees of freedom from 6 to 10 for long-term trends to ensure result robustness. The covariate was also added to the model to adjust for the impact of the COVID-19. All statistical analyses were performed using R software (version 4.4.1), mainly using the “DLNM” package, with *p* < 0.05 considered statistically significant.

## Results

### Descriptive statistics

The statistical summary of meteorological factors, pollutants, and number of outpatient visits were presented in Table 1. The time series distribution of daily air pollutant concentration and meteorological factors were shown in Figure S1 and Figure S2. Following data screening and cleaning, a total of 77,968 outpatient records for children with respiratory disease were obtained from January 1, 2016, to December 31, 2023. Among these records, there were 60,492 related to IRD and 16,997 pertaining to CRD. The time series graph indicates that O_3_ has an overall trend of high concentration in warm season and low concentration in cold season, conversely, other air pollutants generally show lower concentrations during warm seasons but higher levels during cold seasons.

**Table 1:** Statistical summary of air pollutants, meteorological factors and daily number of outpatient visits for RD.

**Variable**	***P*_50_**	***IQR***	***P*_25_**	***P*_75_**	***Mean***	***SD***	***Max***	***Min***
PM_2.5_ (µg/m^3^)	36.0	39.4	21.6	61.0	47.6	37.7	276.2	3.4
PM_10_ (µg/m^3^)	61.6	53.4	39.4	92.8	72.5	45.3	339.2	7.2
O_3_ (µg/m^3^)	83.2	60.6	56.0	116.6	87.3	40.4	212.6	6.0
NO_2_ (µg/m^3^)	26.5	13.4	20.6	34.0	28.3	10.6	82.4	8.2
SO_2_ (µg/m^3^)	7.6	4.2	6.4	10.6	9.2	4.6	45.6	3.8
CO (mg/m^3^)	0.8	0.4	0.6	1.0	0.8	0.3	2.6	0.2
TEMP (°C)	17.5	14.7	9.7	24.4	17.0	8.4	32.9	−3.8
RH (%)	76.0	21.9	65.3	87.2	75.6	14.5	100.0	30.3
WS (m/s)	1.8	0.7	1.4	2.1	1.8	0.5	4.2	0.4
RD number	24.0	21.0	15.0	36.0	26.7	16.9	149.0	0.0
IRD number	19.0	15.0	12.0	27.0	20.7	13.8	144.0	0.0
CRD number	4.0	6.0	2.0	8.0	5.8	5.1	45.0	0.0

PM_2.5_: particulate matter smaller than 2.5 µm, PM_10_: particulate matter smaller than 10 µm, O_3_: ozone, NO_2_: nitrogen dioxide, SO_2_: sulfur dioxide, CO: carbon monoxide, TEMP: average daily temperature, RH: average relative humidity, WS: daily average wind speed, RD: respiratory disease, IRD: infectious respiratory diseases, CRD: chronic respiratory disease, IQR: interquartile range, Max: maximal value, Min: minimal value.

The median of air pollutants was as follows: PM_2.5_ for 36.0 µg/m^3^; PM_10_ for 61.6 µg/m^3^; O_3_ for 83.2 µg/m^3^; NO_2_ for 26.5 µg/m^3^; SO_2_ for 7.6 µg/m^3^ and CO for 0.8 mg/m^3^. The daily mean values of temperature, relative humidity and wind speed were 17.0 °C, 75.6% and 1.8 m/s respectively.

**Fig. 1:**
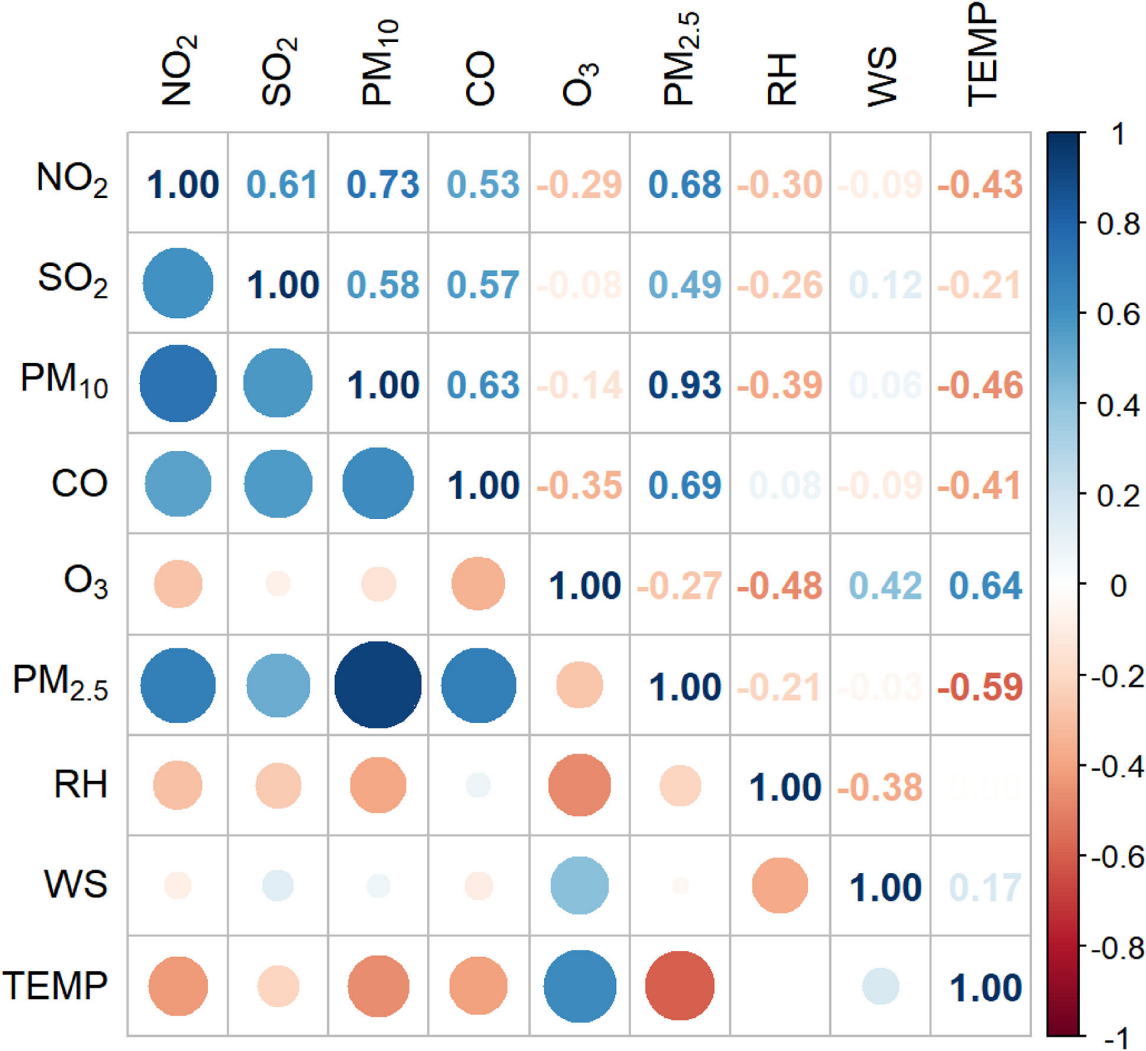
The correlation between air pollutants and meteorological indicators.

The Spearman correlation coefficients between meteorological factors and air pollutants were shown in Fig. 1, the biggest one was 0.64, which showed the correlation between O_3_ and TEMP. Considering the correlation and collinearity between pollutants and meteorological factors, the variance expansion factor of each single pollutant model was calculated and no model showed serious multicollinearity.

### Relationship between air pollutants and outcomes

**Fig. 2:**
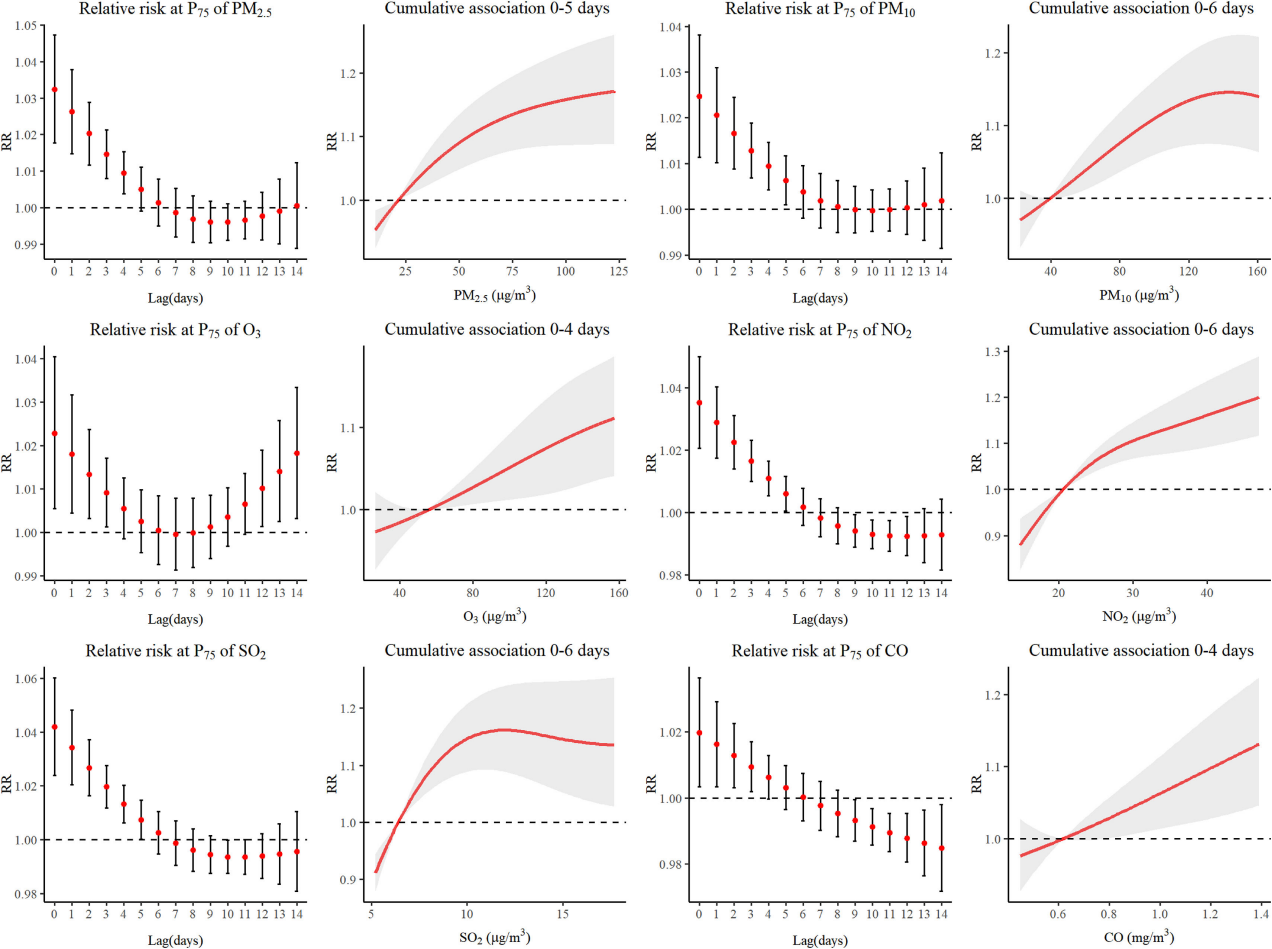
Relative risk and cumulative association with different lag days of air pollutants on RD.

Fig. 2 illustrated the delayed impacts and estimated cumulative association of air pollutants on the number of daily outpatients for respiratory diseases in children. The nonlinear relationship was found between all air pollutants and outcome. The maximum lag day for air pollutant was the day when the RR value has no statistical significance, lag4 for O_3_ and CO, lag5 for PM_2.5_, and lag6 for PM_10_, NO_2_ and SO_2_. The relative risk value of each pollutant was highest at lag 0 and exhibit a gradual decline as lag days increased. The maximum RR values along with corresponding confidence intervals were presented below: PM_2.5_ with RR 1.032 (95% CI: 1.018–1.047); PM_10_ with RR 1.025 (95%CI: 1.011–1.038); O_3_ with RR 1.023 (95% CI: 1.005–1.041); NO_2_ with RR 1.035 (95% CI: 1.021–1.050); SO_2_ with RR 1.042 (95% CI: 1.024–1.060) and CO with RR 1.020 (95% CI: 1.003–1.036). Figure S3 illustrated the exposure-response curve for lag0 corresponding to the day of maximum pollutant impact value.

**Fig. 3:**
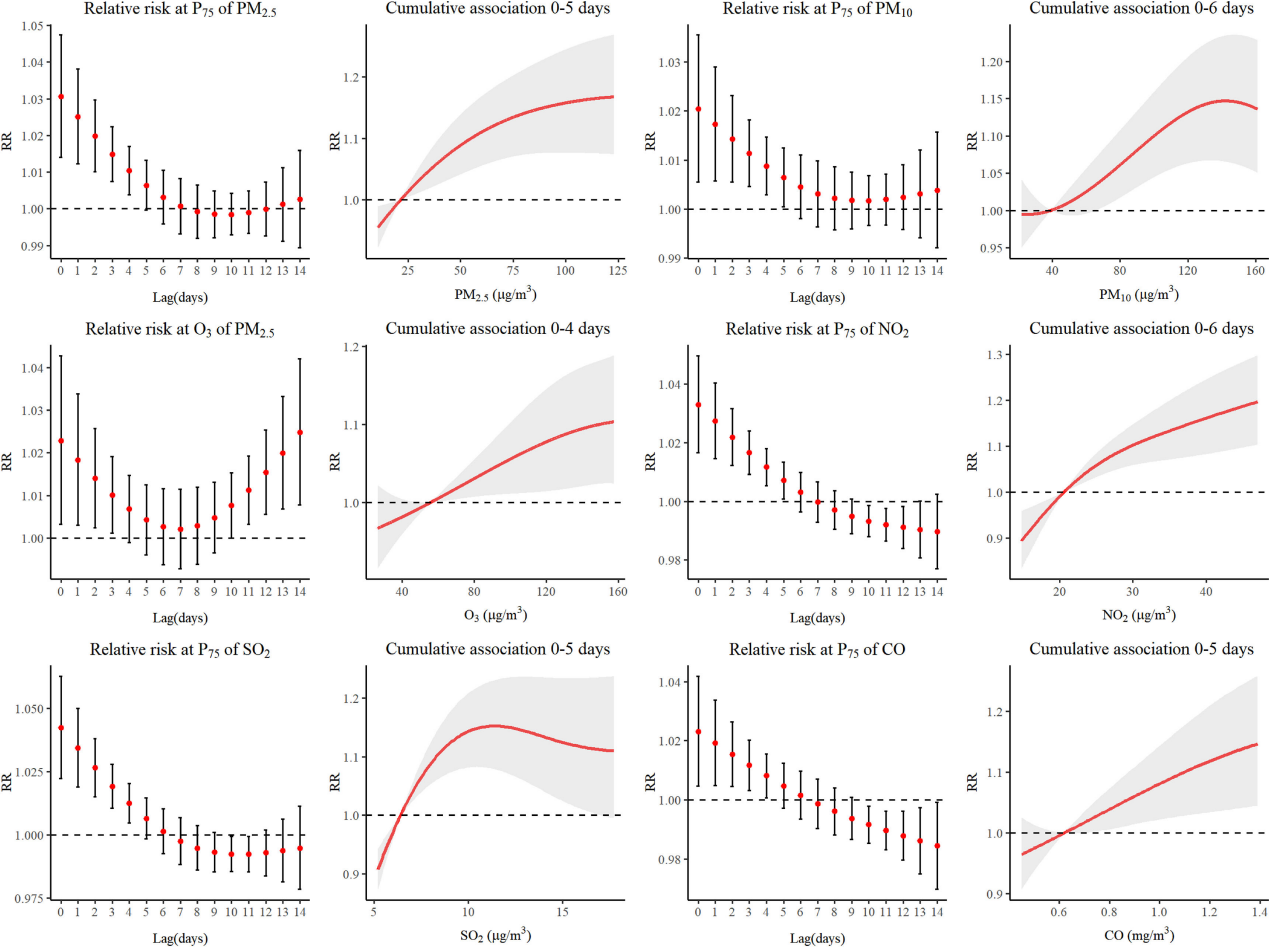
Relative risk and cumulative association with different lag days of air pollutants on IRD.

Fig. 3 presented the analysis results of infectious respiratory diseases. Because of the amount of this subcategory account for a large proportion of respiratory disease, the impacts caused by air pollutants were similar. The nonlinear relationship was found between all air pollutants and outcome and the RR values were gradually decreased with the increase of lag days. The maximum lag days of each pollutant were 4–6 day.

**Fig. 4:**
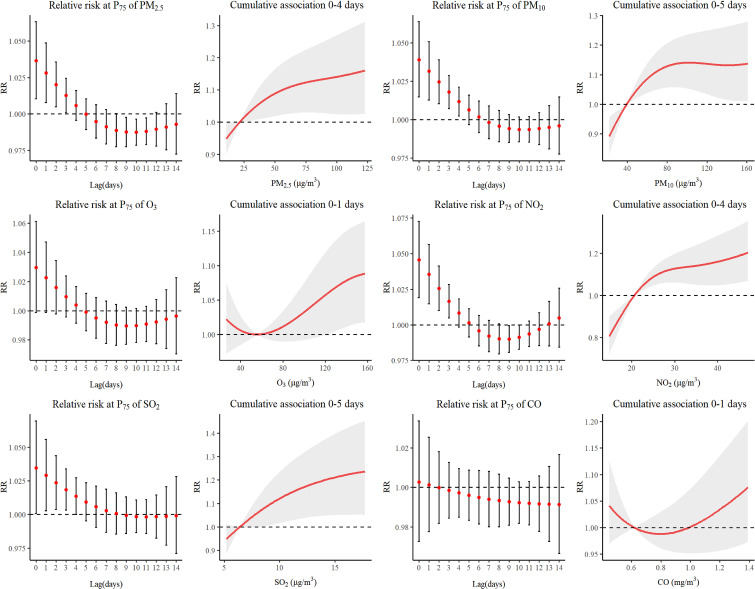
Relative risk and cumulative association with different lag days of air pollutants on CRD.

Fig. 4 detailed the analytical findings about chronic respiratory disease in children. The maximum of RR value of PM_2.5_ was 1.036 (95%CI: 1.010–1.063), PM_10_ was 1.039 (95%CI: 1.014–1.064), NO_2_ was 1.046 (95%CI: 1.019–1.073) and SO_2_ was 1.035 (95%CI: 1.001–1.070) The lag impacts of PM_2.5_ and NO_2_ were observed to persist until lag4, while the impacts of PM_10_ and SO_2_ lasted until lag5.

### Subgroup analysis

**Fig. 5:**
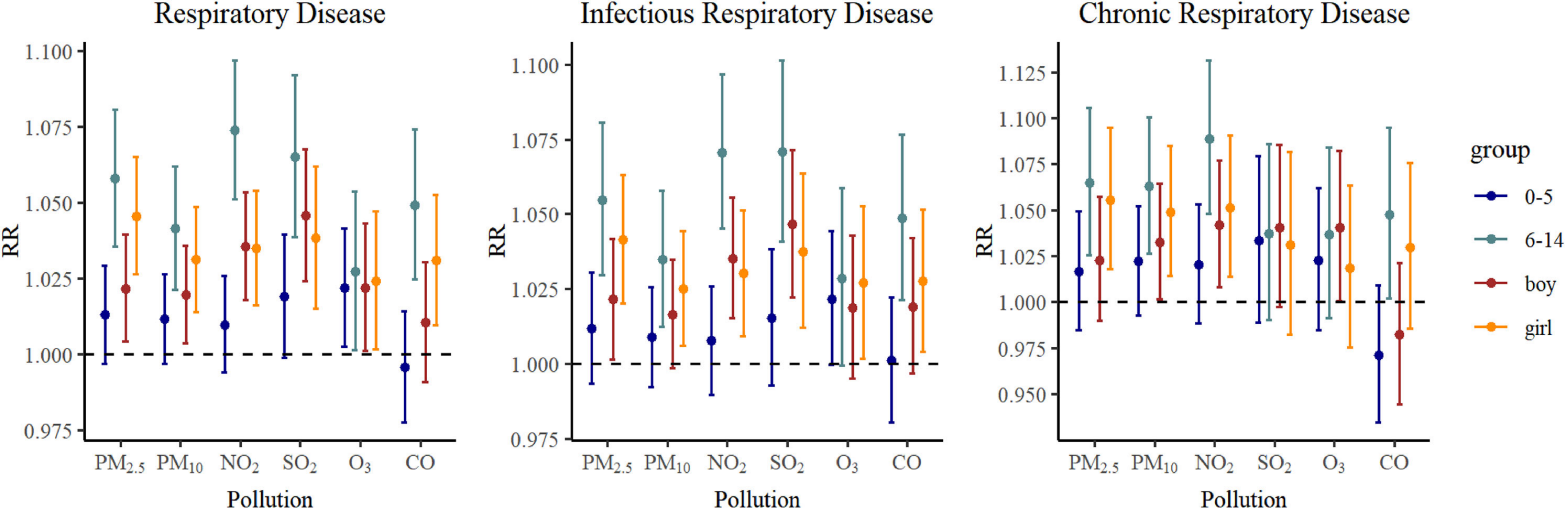
Relative risks (95% CI) in lag0 of RD, IRD and CRD in subgroup analyses.

**Fig. 6:**
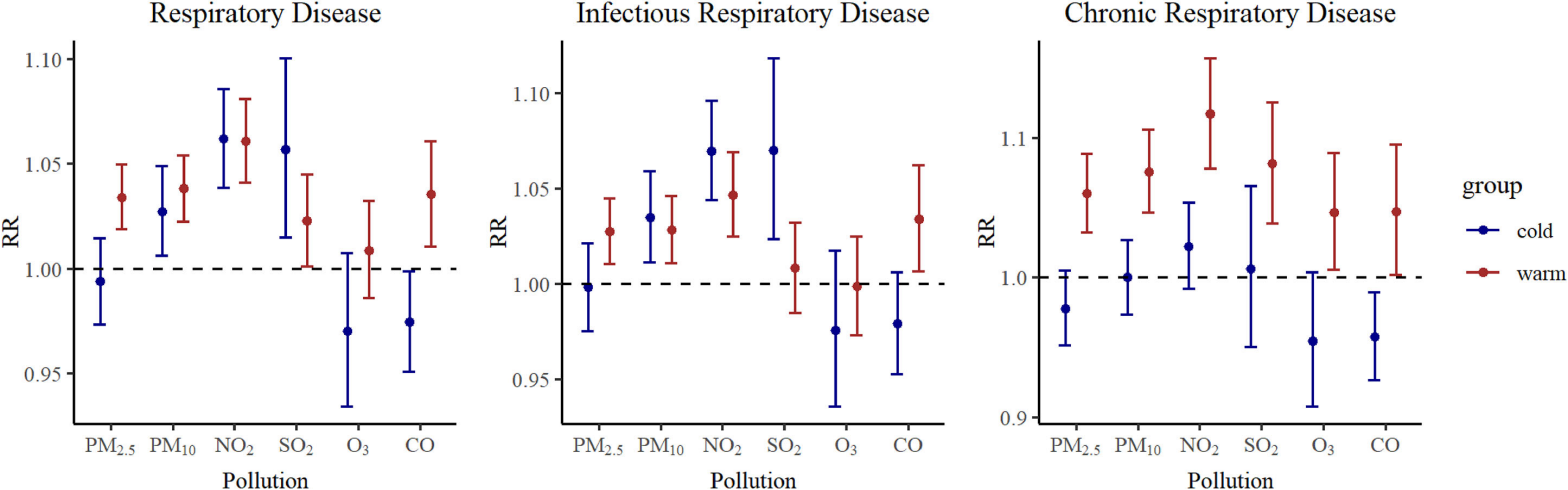
Relative risks (95% CI) in lag0 of RD, IRD and CRD in different season.

Subgroup analyses were conducted based on age, gender and season. The overall exposure-response relationships between the six air pollutants and respiratory diseases exhibited similar trends for both boy and girl, with no statistically significant differences observed (Table S1, Table S2 and Table S3). Participants were categorized into preschoolers aged 0–5 years and school-aged children from 6–14 years. Notably, school-aged children demonstrated a higher susceptibility to air pollutants compared to preschoolers, with the difference reaching statistical significance. Fig. 5 showed relative risks (RR 95% CI) in lag0 of children respiratory disease, infectious respiratory disease and chronic respiratory disease outpatient visits associated with air pollutants in subgroup analyses. For chronic respiratory diseases, the impacts of air pollutants in the cold season and the warm season were different, and the health risks of six pollutants are stronger in the warm season. Fig. 6 showed relative risks (RR 95% CI) in warm and cold season.

### Sensitivity analysis

**Fig. 7:**
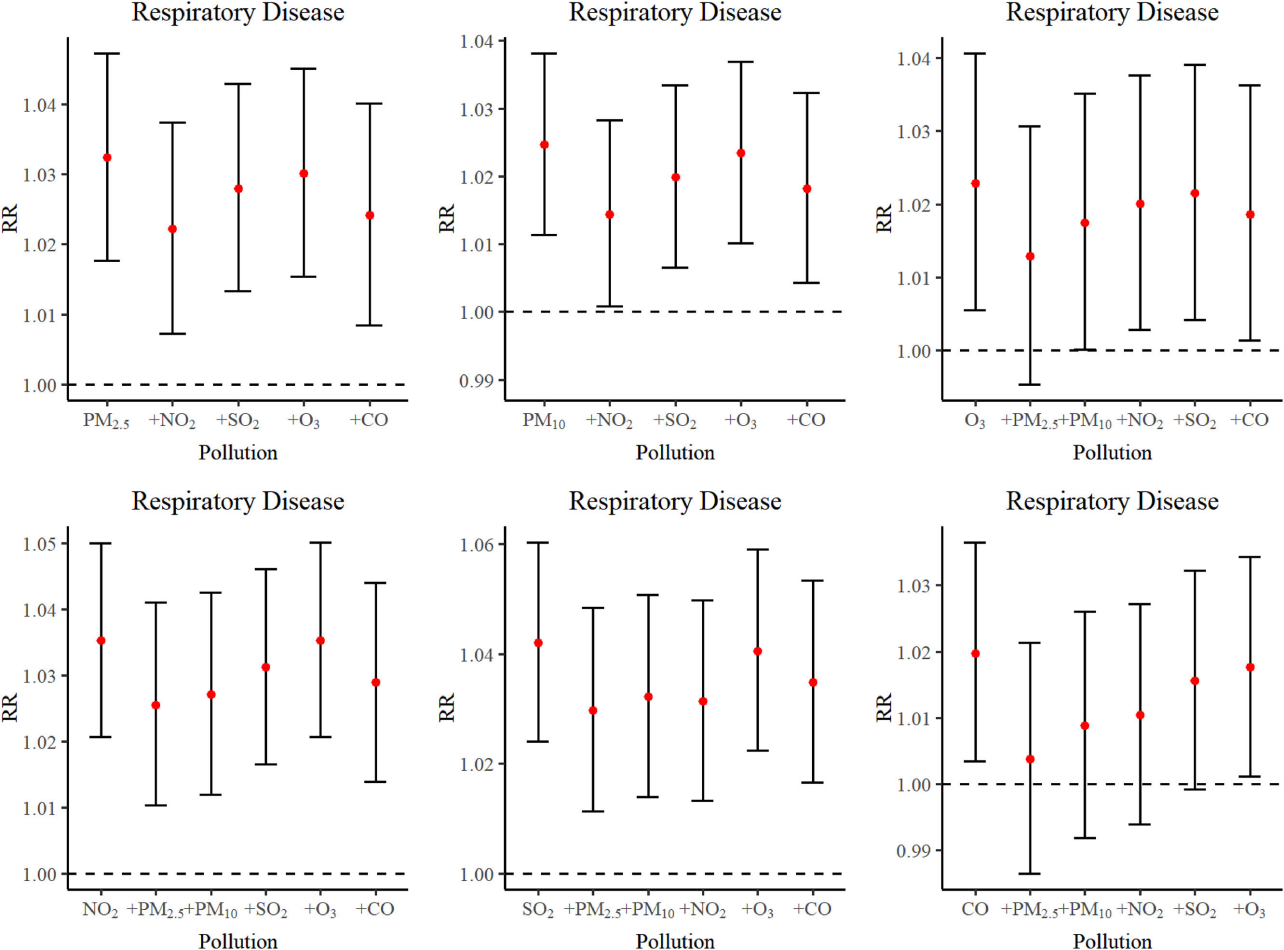
Relative risks (95% CI) in lag0 of RD in dual pollutant model.

**Fig. 8:**
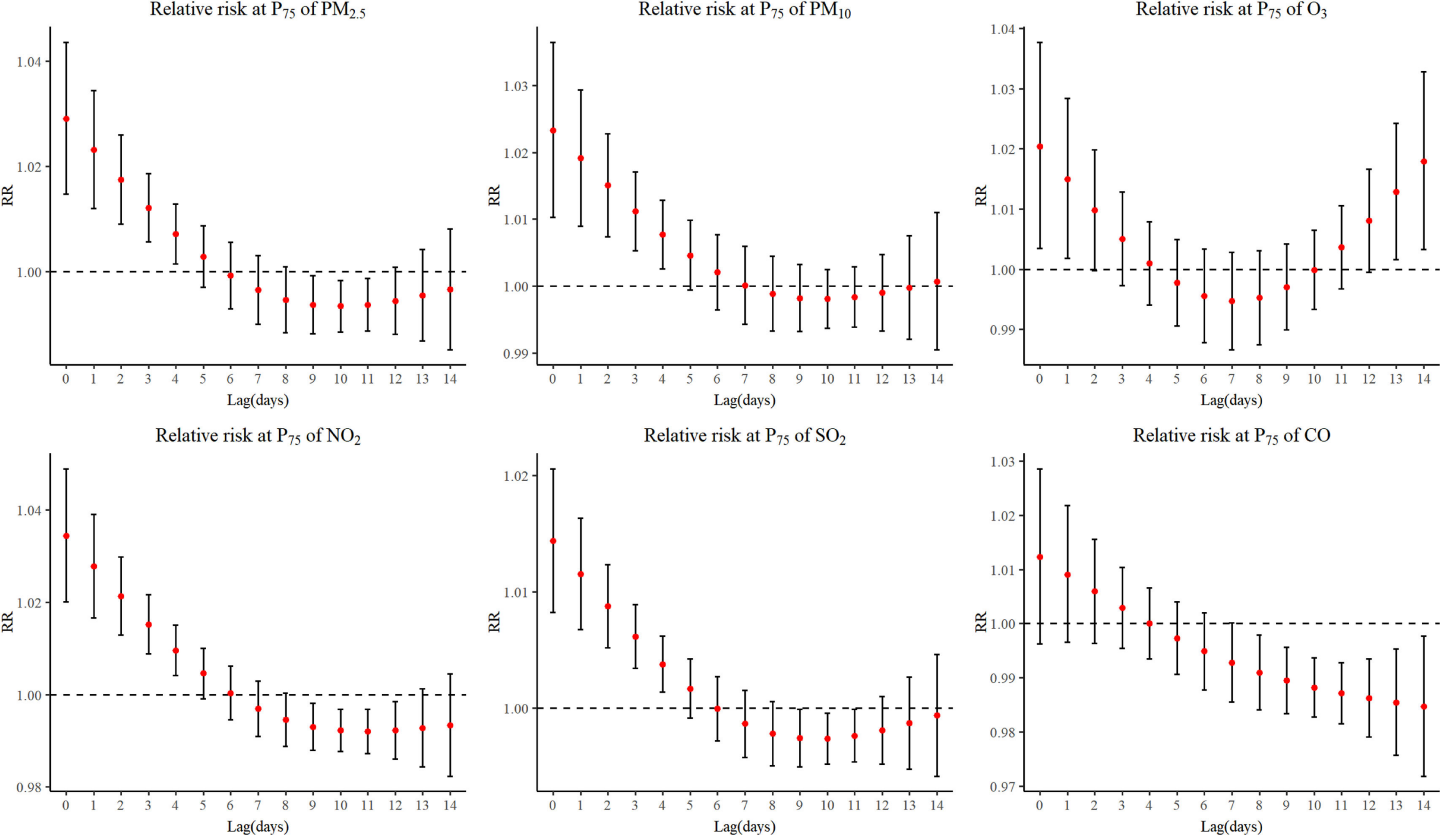
Relative risks (95% CI) in lag0 of RD after adjusting COVID-19.

Table S4 presented a summary of results from the dual pollutant model after incorporating an additional air pollutant into the base model. The findings indicated that statistical outcomes remained stable after the adjustment. Fig. 7, Figure S4 and Figure S5 displayed relative risk (RR 95% CI) in lag0 of dual pollutant model. Table S5 illustrated the impact of pollutants while adjusting the df of time, which revealed no significant change of RR values, confirming that statistical results remained consistent. Fig. 8 detailed the analytical results after adjusting COVID-19. Except for CO, the adjusted statistical results remained stable.

## Discussion

We conducted a time series analysis utilizing 77,968 outpatient records of children with respiratory diseases from 2016 to 2023 in Yichang, China. Our findings indicated that current exposure (lag0) to pollutants caused the maximum impact value. As the number of lag days increased, the impact gradually diminished, notably, the lag impact persisted for approximately 4–6 days. Furthermore, health impact attributable to air pollutants varied across different types of respiratory diseases in children.

Air pollutants such as PM, SO_2_, NO_2_, CO, and O_3_ originating from industry and other sources have been recognized as contributors to respiratory disease [17–21]. Due to the growth and development characteristics of children, they are particularly susceptible to air pollutants [22]. Previous studies have examined the impacts of air pollution exposure on the incidence of respiratory diseases in children. A study of 593 children in New York City indicated that living in neighborhoods characterized by higher levels of air pollution, dense traffic and industrial facilities may contribute to respiratory diseases [23]. Certain epidemiological investigations have also revealed that some demographic groups, including children, the elderly, individuals with chronic illnesses, and low-income populations, exhibited higher sensitivity to air pollution [24–26]. Currently, research on the short-term impacts of air pollution on children outpatient visits is quite limited.

Overall, our findings were similar to other studies that have focused on children, however, the majority of these studies concentrated on hospitalized pediatric populations or specific disease. For example, a study conducted in Taipei found that the increase in PM_2.5_ at relatively low levels could lead to a rise in current visits to pediatric respiratory clinics [27]. A study in Brazil, showed that high levels of O_3_ and SO_2_ could lead to more frequent hospital visits for pneumonia and influenza [28]. A study showed that short-term increases in NO_2_, PM_2.5_, and CO were positively associated with outpatient visits for acute bronchitis among children [29]. A large study conducted in 25 districts in seven cities in northeast China looked specifically at the impacts of outdoor and indoor air pollution on asthma and asthma-related symptoms in children aged 6 to 13 years [30]. Our study employed outpatient data with a variety of conditions which included both mild and severe cases among children. It is possible to provide a more comprehensive assessment of the impact of air pollutants on children respiratory disease. Our results showed that air pollution such as PM_2.5_, PM_10_, O_3_, NO_2_, SO_2_, CO had significant influence on outpatient visits for respiratory diseases in children and the risk increases with the rise of pollutant concentration. The results were demonstrated to be stable through sensitivity analysis.

The effects of pollutants on infectious and chronic respiratory diseases were different because of different pathogenesis. For infectious respiratory diseases, air pollutants carrying with harmful substances such as fungi, bacteria and viruses entered from mouth and nose then deposited in the airway and alveolar for a long time causing a toxic effect [31, 32]. Chronic respiratory diseases were exacerbated by the synergistic impacts of respiratory inflammation and oxidative stress induced by air pollutants. In our findings, all six air pollutants had adverse impacts on infectious respiratory disease, but the impacts of CO and O_3_ were relatively weak. As for chronic respiratory disease, PM_2.5_, PM_10_, NO_2_ and SO_2_, had adverse impacts, furthermore, the impacts of those four were smaller compared to their influence on IRD. O_3_ and CO did not have statistical significance impacts to CRD. The results of the dual pollutant model showed that the impacts of O_3_ and CO were weakened when affected by other factors, which may be the reason for their relatively small impact. Notably, the exposure-response curve of PM_2.5_, PM_10_, NO_2_ and SO_2_ for infectious respiratory disease reached approximated threshold, tending to be stabilized. One possible reason was the saturation mechanism, physiological and biochemical reaction will not increase when cells reach the saturation dose. However, the exposure-response curve for chronic respiratory diseases is different which had a wider confidence interval. One potential explanation was the small sample size under high concentration exposure and the number of patients with chronic diseases was less.

Our findings indicated that the lag impacts of various pollutants range from 4 to 6 days and prior research corroborated our findings. For example, a study conducted in Ganzhou, China, demonstrated that a 6.9% increase in the RR of respiratory disease for rising in SO_2_ and lag impacts lasted for 5 days [33]. Yang concluded that O_3_ level 2–5 days before admission was associated with hospitalizations for children respiratory illness [34]. In Lanzhou, China, short-term exposure to PM_2.5_, PM_10_, NO_2_, and CO posed greater risks with lag impacts lasting for 7 days [14]. Although the number of lag days varied from studies, this may be due to geographical and climatic factors affecting the dispersal of pollutants. Subgroup analyses indicated that school-aged children exhibited a higher relative risk compared to preschool-aged children and some studies also have corroborated this notion [29, 35]. This may be attributed to the fact that school-aged children spent more chance outdoors, encountered more complex environments, and were exposed to elevated levels of pollutants. In the warm season, air pollutants have a greater impact on children’s chronic respiratory diseases. During the warm season, children tend to spend significantly more time outdoors, meanwhile, people often open windows wide for ventilation. As a result, children are more prone to environmental exposure. The warm season brings an abundance of allergens, like pollen from blooming plants and dust mites thriving in the warmer, often more humid conditions. These allergens interact with air pollutants causing children with chronic respiratory diseases are prone to relapse.

Our findings carried significant public health implications. First, air pollution had attracted a lot of attention due to its adverse health impacts, and our study provided evidence that air pollution was associated with increased outpatient visits among children and revealed that the impact varied across different types of respiratory diseases, thereby facilitating targeted protective measures. Second, we quantified the exposure-response relationship, delayed impacts, and cumulative impacts of short-term air pollution exposure on outpatient visits for pediatric respiratory diseases, which can inform and enhance control efforts based on monitored air quality data. A notable strength of this study was that it explored the impacts of six distinct air pollutants on outpatient visits for different types of respiratory diseases in children, and highlighting differences between disease types as well as age subgroups. Additionally, the impacts of meteorological factors, time long-term trends, as well as the hysteresis impacts were taken into account, which ensured the results aligned more closely with real-world conditions. Sensitivity analyses further confirmed the robustness of our results. However, several limitations need to be considered. We employed average monitoring data from five pollutant monitoring sites as a proxy for daily exposure, which may not be able to accurately estimate exposure. Furthermore, our analysis was limited to a single hospital in Yichang, raising questions about the generalizability of our findings to other regions or populations. Lastly, although we have adjusted for some confounding factors, there may still be other confounding factors that have an impact to some extent. Because outpatient records were aggregated into time series, the individual clinical diagnosis indicators of disease were not included in the model.

## Conclusion

In our study, PM_2.5_, PM_10_, NO_2_, and SO_2_ were found to exert a more significant influence on outpatient visits among children with respiratory diseases, whereas O_3_ and CO demonstrated comparatively minor impacts. It was recommended that greater emphasis should be placed on these pollutants in environmental monitoring, with timely warnings and targeted interventions implemented to reduce air pollutant concentrations. The disparity in air pollutant exposure between preschool and school-age children indicated that students face a significant risk of exposure, necessitating adjustments to outdoor activity schedules based on air quality conditions, along with the implementation of personal protective measures. Therefore, to safeguard the health of Chinese children, effective intervention strategies should be implemented to enhance control measures for ambient air pollutants while prioritizing protective efforts aimed at this demographic in order to mitigate the burden of respiratory diseases.

## Abbreviations

ICD-10: International Classification of Diseases
TEMP: average daily temperature
RH: average relative humidity
WS: daily average wind speed
RD: children respiratory disease
IRD: children infectious respiratory diseases
CRD: children chronic respiratory disease
DLNM: distributed lag nonlinear model

## Supplementary information

**Additional file 1:** **Figure S1.** Time series distribution air pollutants. **Figure S2.** Time series distribution of meteorological factors. **Figure S3.** A: E-R curves of air pollutants and children respiratory disease outpatient visits with reference air pollutant concentrations of the 25^th^ percentile. B: E-R curves of air pollutants and children infectious respiratory disease outpatient visits with reference air pollutant concentrations of the 25^th^ percentile. C: E-R curves of air pollutants and children chronic respiratory disease outpatient visits with reference air pollutant concentrations of the 25^th^ percentile. **Figure S4.** Relative risks (95% CI) in lag0 of children infectious respiratory disease outpatient visits associated with air pollutants, comparing the 75th percentile to the 25th percentile in dual pollutant model. **Figure S5.** Relative risks (95% CI) in lag0 of children chronic respiratory disease outpatient visits associated with air pollutants, comparing the 75th percentile to the 25th percentile in dual pollutant model. **Table S1.** The results of subgroup analyses in children respiratory disease. **Table S2.** The result of subgroup analyses in children infectious respiratory disease. **Table S3.** The result of subgroup analyses in children chronic respiratory disease. **Table S4.** Sensitivity analyses results of dual pollution model. **Table S5.** Sensitivity analyses results of changing degree of freedom.

## Declarations

### Availability of data and material

Data cannot be shared for privacy or ethical reasons.
