# Supplementary material for: Impacts of short-term exposure to ambient air pollutants on outpatient visits for respiratory diseases in children: a time series study in Yichang, China
Source: Environ Health Prev Med. 2025 Mar 12;30:16. doi: 10.1265/ehpm.24-00373 (PMC11925708; doi:10.1265/ehpm.24-00373)
Supplement: Supplementary file 1 — Additional file 1: Figure S1. Time series distribution air pollutants. Figure S2. Time series distribution of meteorological factors. Figure S3. A: E-R curves of air pollutants and children respiratory disease outpatient visits with reference air pollutant concentrations of the 25th percentile. B: E-R curves of air pollutants and children infectious respiratory disease outpatient visits with reference air pollutant concentrations of the 25th percentile. C: E-R curves of air pollutants and children chronic respiratory disease outpatient visits with reference air pollutant concentrations of the 25th percentile. Figure S4. Relative risks (95% CI) in lag0 of children infectious respiratory disease outpatient visits associated with air pollutants, comparing the 75th percentile to the 25th percentile in dual pollutant model. Figure S5. Relative risks (95% CI) in lag0 of children chronic respiratory disease outpatient visits associated with air pollutants, comparing the 75th percentile to the 25th percentile in dual pollutant model. Table S1. The results of subgroup analyses in children respiratory disease. Table S2. The result of subgroup analyses in children infectious respiratory disease. Table S3. The result of subgroup analyses in children chronic respiratory disease. Table S4. Sensitivity analyses results of dual pollution model. Table S5. Sensitivity analyses results of changing degree of freedom. [file ehpm-30-016-s001.docx]

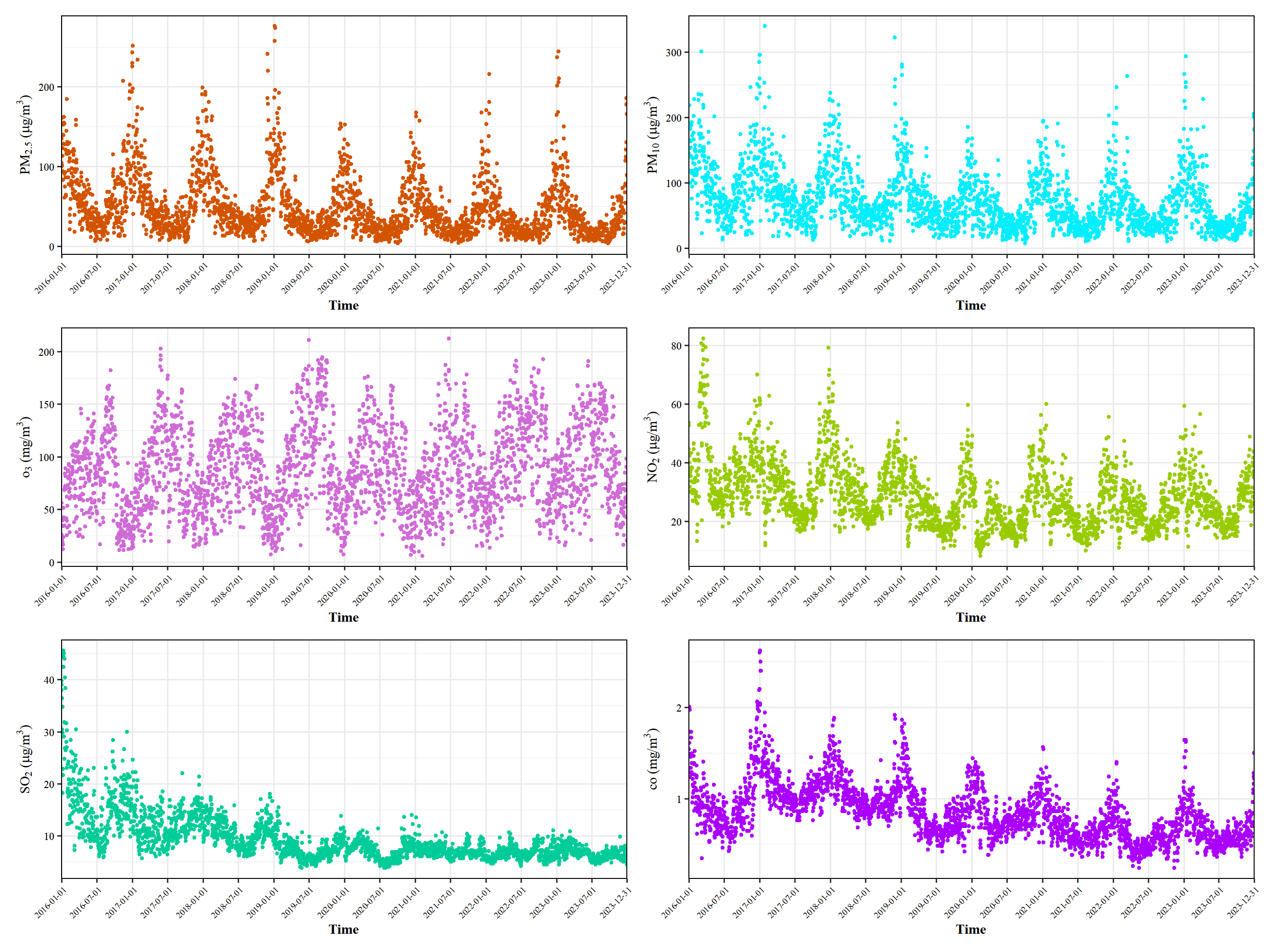


Figure S1. Time series distribution air pollutants.


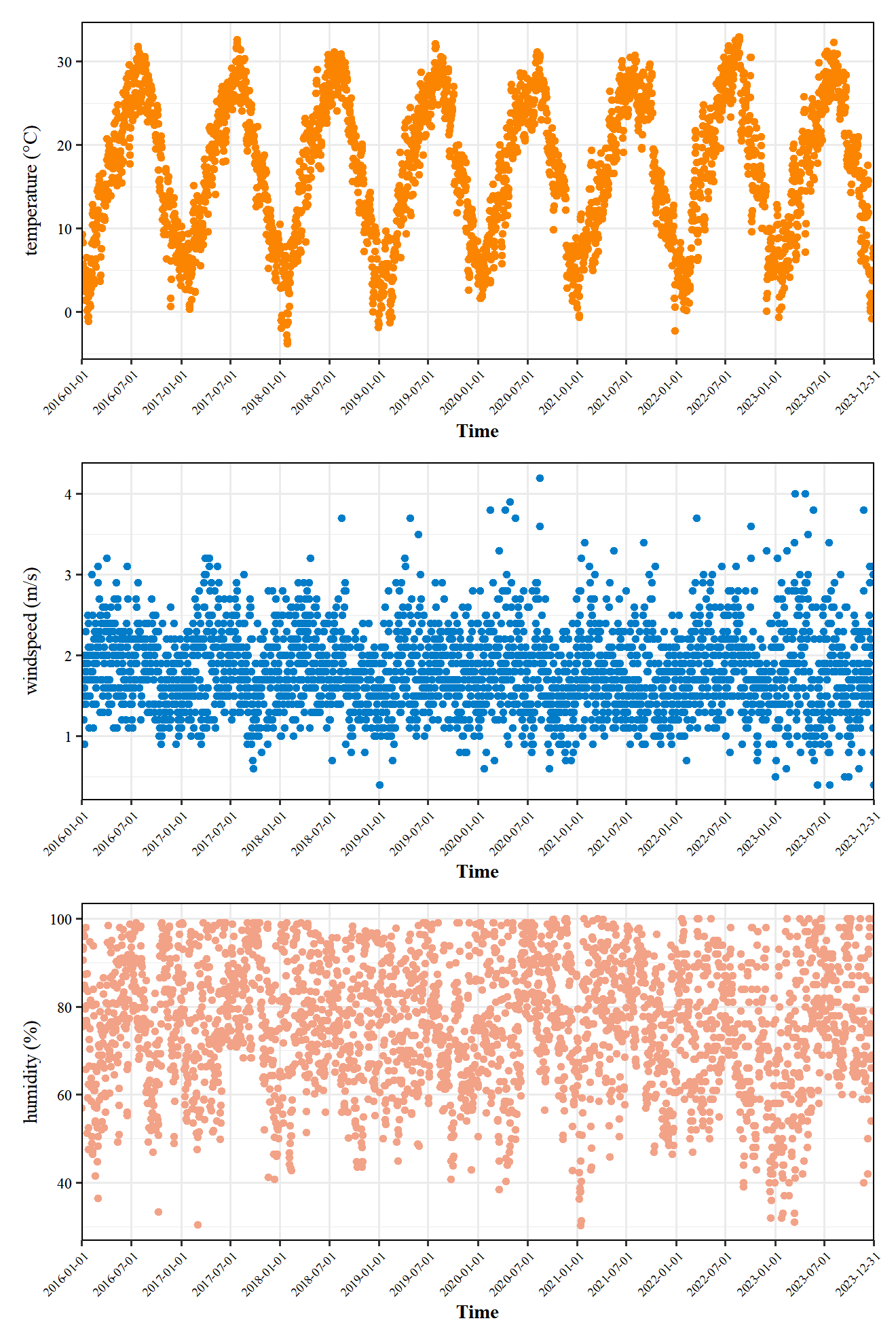


Figure S2. Time series distribution of meteorological factors.
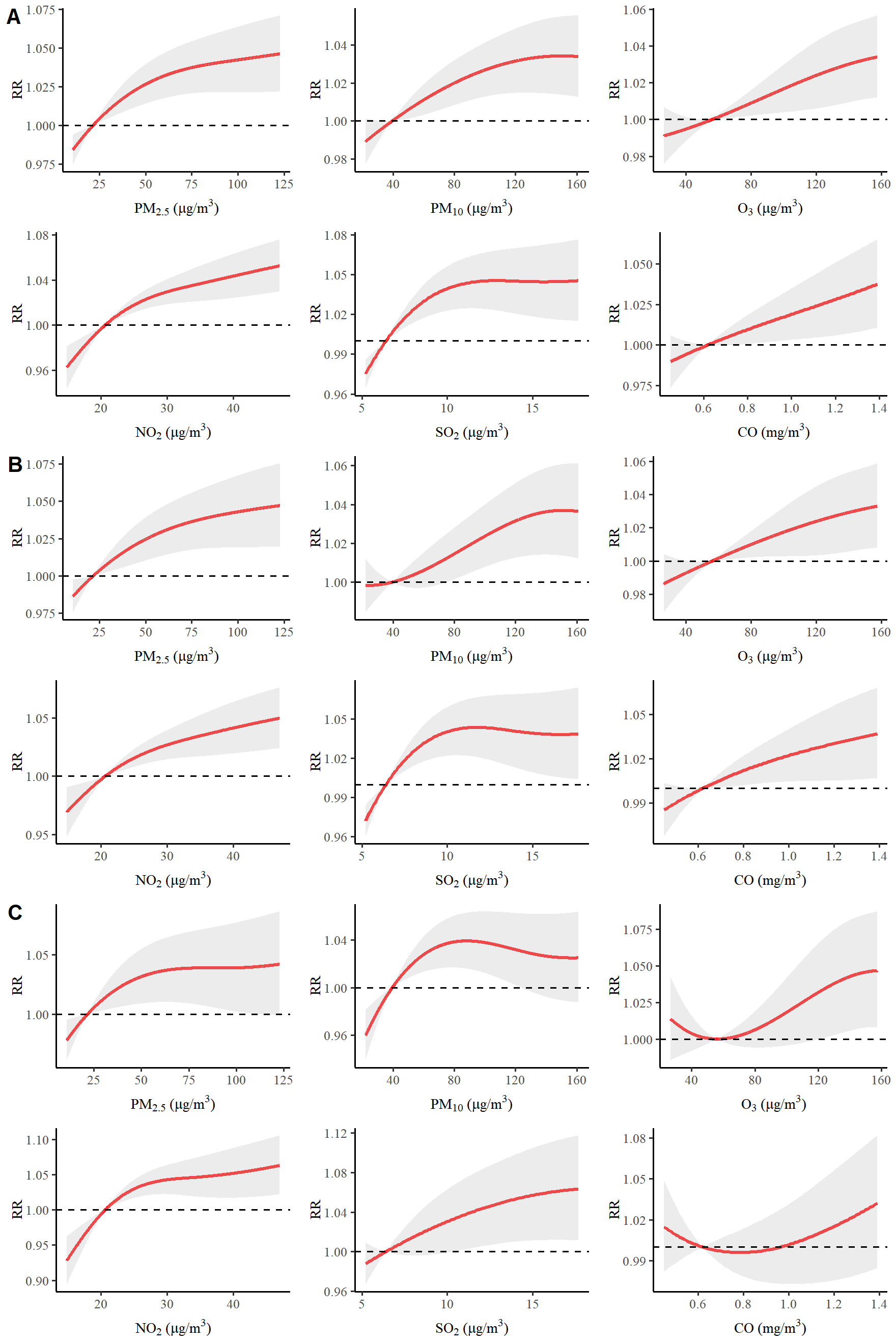


Figure S3. A: E-R curves of air pollutants and children respiratory disease outpatient visits with reference air pollutant concentrations of the 25^th^ percentile. B: E-R curves of air pollutants and children infectious respiratory disease outpatient visits with reference air pollutant concentrations of the 25^th^ percentile. C: E-R curves of air pollutants and children chronic respiratory disease outpatient visits with reference air pollutant concentrations of the 25^th^ percentile.
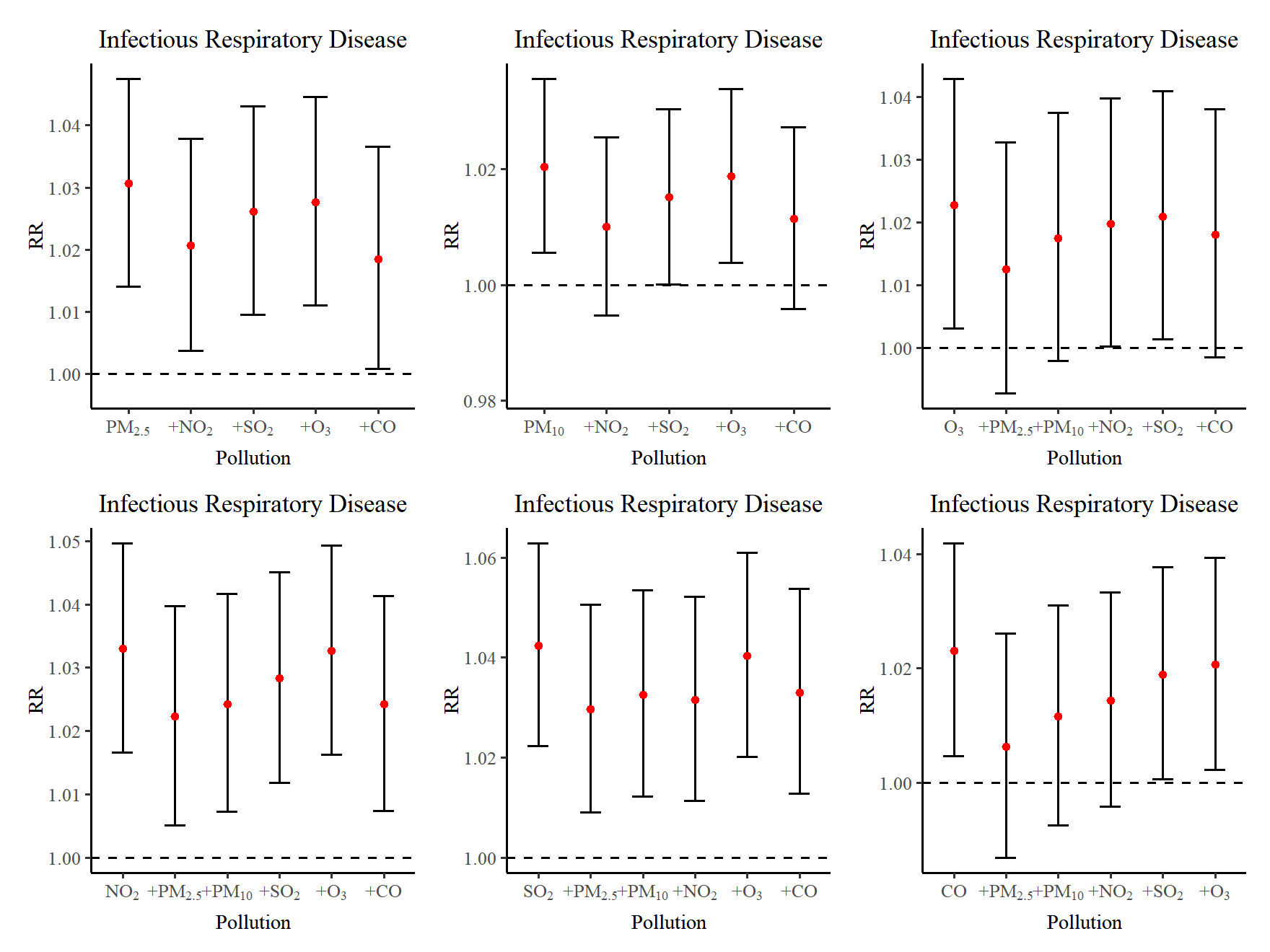


Figure S4. Relative risks (95% CI) in lag0 of children infectious respiratory disease outpatient visits associated with air pollutants, comparing the 75th percentile to the 25th percentile in dual pollutant model.


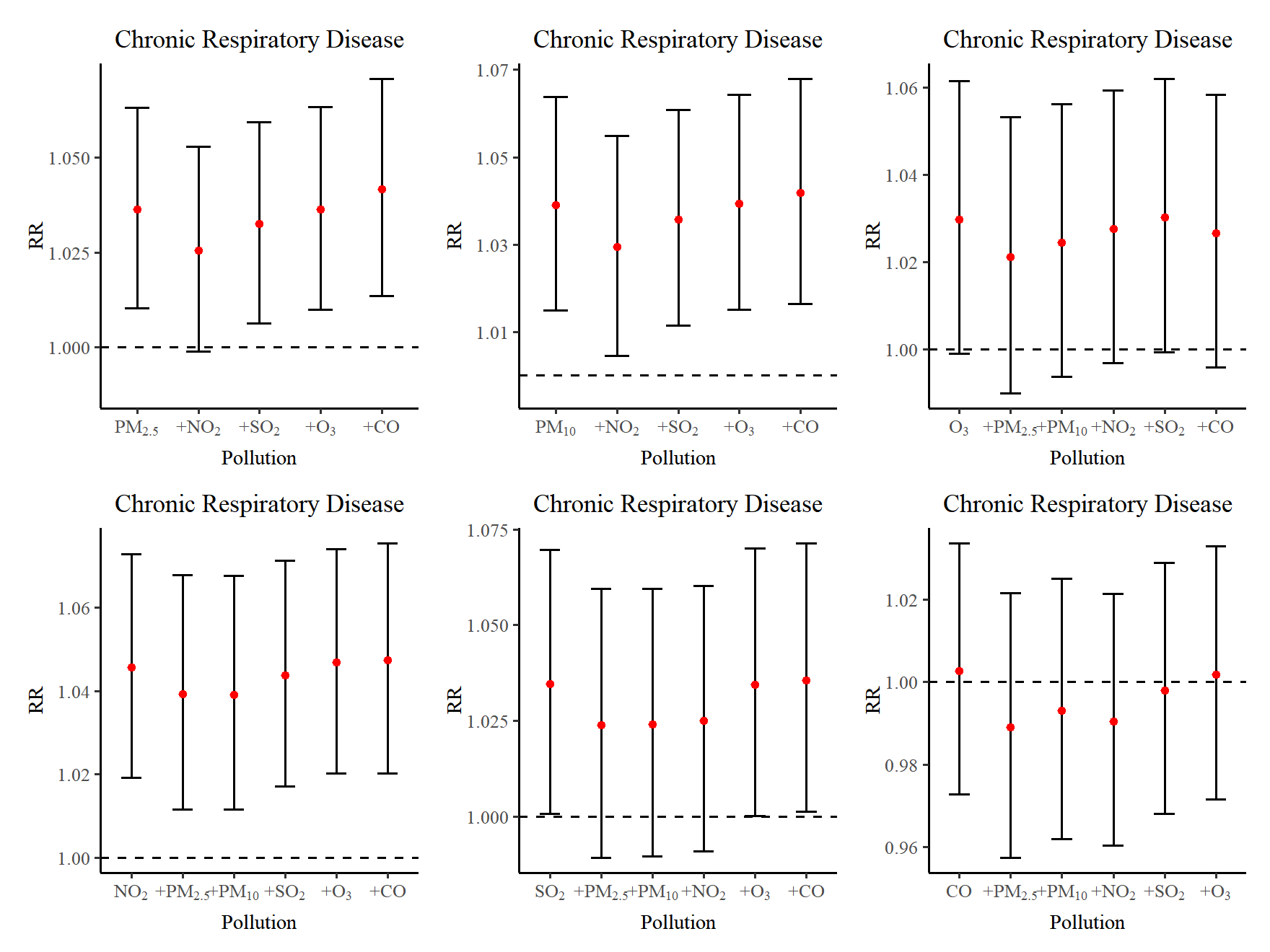


Figure S5. Relative risks (95% CI) in lag0 of children chronic respiratory disease outpatient visits associated with air pollutants, comparing the 75th percentile to the 25th percentile in dual pollutant model.

Table S1. The results of subgroup analyses in children respiratory disease.

| **Pollutants** | **Days** | **0-5** | **6-14** | **boy** | **girl** |
| --- | --- | --- | --- | --- | --- |
| **PM_2.5_** | Lag0 | 1.013(0.997,1.029) | 1.058(1.036,1.081) | 1.022(1.004,1.039) | 1.046(1.026,1.065) |
|  | Lag1 | 1.011(0.998,1.024) | 1.046(1.029,1.063) | 1.017(1.003,1.031) | 1.038(1.023,1.053) |
|  | Lag2 | 1.009(1.000,1.019) | 1.034(1.022,1.047) | 1.012(1.002,1.022) | 1.030(1.019,1.042) |
|  | Lag3 | 1.007(1.000,1.015) | 1.024(1.014,1.034) | 1.008(1.000,1.016) | 1.023(1.014,1.032) |
|  | Lag4 | 1.005(0.999,1.012) | 1.014(1.006,1.023) | 1.004(0.997,1.011) | 1.016(1.009,1.024) |
|  | Lag5 | 1.004(0.997,1.01) | 1.006(0.997,1.015) | 1.000(0.993,1.008) | 1.011(1.003,1.018) |
| **PM_10_** | Lag0 | 1.012(0.997,1.026) | 1.041(1.021,1.062) | 1.019(1.004,1.036) | 1.031(1.014,1.049) |
|  | Lag1 | 1.010(0.998,1.021) | 1.034(1.019,1.050) | 1.016(1.003,1.028) | 1.027(1.013,1.040) |
|  | Lag2 | 1.008(0.999,1.017) | 1.027(1.015,1.039) | 1.012(1.003,1.022) | 1.022(1.012,1.032) |
|  | Lag3 | 1.006(0.999,1.013) | 1.021(1.012,1.030) | 1.009(1.002,1.016) | 1.018(1.010,1.026) |
|  | Lag4 | 1.004(0.998,1.01) | 1.015(1.007,1.023) | 1.006(1.000,1.012) | 1.014(1.007,1.020) |
|  | Lag5 | 1.003(0.997,1.009) | 1.01(1.002,1.0180) | 1.003(0.997,1.010) | 1.010(1.003,1.017) |
|  | Lag6 | 1.001(0.995,1.008) | 1.006(0.997,1.014) | 1.001(0.994,1.008) | 1.007(0.999,1.014) |
| **O_3_** | Lag0 | 1.022(1.002,1.042) | 1.027(1.001,1.054) | 1.022(1.001,1.043) | 1.024(1.002,1.047) |
|  | Lag1 | 1.016(1.001,1.031) | 1.024(1.003,1.044) | 1.017(1.001,1.034) | 1.019(1.002,1.037) |
|  | Lag2 | 1.010(0.999,1.022) | 1.020(1.005,1.035) | 1.013(1.000,1.025) | 1.015(1.001,1.028) |
|  | Lag3 | 1.005(0.996,1.014) | 1.017(1.005,1.028) | 1.009(0.999,1.018) | 1.010(1.000,1.021) |
|  | Lag4 | 1.001(0.993,1.008) | 1.014(1.003,1.024) | 1.005(0.997,1.013) | 1.007(0.997,1.016) |
| **NO_2_** | Lag0 | 1.01(0.994,1.0260) | 1.074(1.051,1.097) | 1.036(1.018,1.053) | 1.035(1.016,1.054) |
|  | Lag1 | 1.008(0.995,1.020) | 1.060(1.042,1.078) | 1.029(1.015,1.042) | 1.029(1.014,1.044) |
|  | Lag2 | 1.006(0.997,1.016) | 1.047(1.033,1.060) | 1.022(1.011,1.032) | 1.023(1.012,1.035) |
|  | Lag3 | 1.004(0.997,1.012) | 1.034(1.024,1.044) | 1.015(1.008,1.023) | 1.018(1.009,1.026) |
|  | Lag4 | 1.003(0.996,1.009) | 1.022(1.014,1.031) | 1.010(1.003,1.016) | 1.013(1.005,1.020) |
|  | Lag5 | 1.001(0.995,1.007) | 1.013(1.004,1.021) | 1.004(0.998,1.011) | 1.008(1.001,1.015) |
|  | Lag6 | 0.999(0.993,1.006) | 1.004(0.995,1.013) | 1.000(0.993,1.007) | 1.004(0.996,1.011) |
| **SO_2_** | Lag0 | 1.019(0.999,1.040) | 1.065(1.039,1.092) | 1.046(1.024,1.068) | 1.038(1.015,1.062) |
|  | Lag1 | 1.014(0.999,1.030) | 1.054(1.034,1.075) | 1.037(1.021,1.054) | 1.031(1.013,1.049) |
|  | Lag2 | 1.010(0.998,1.022) | 1.044(1.029,1.059) | 1.029(1.017,1.042) | 1.024(1.011,1.038) |
|  | Lag3 | 1.006(0.997,1.015) | 1.034(1.023,1.046) | 1.021(1.012,1.031) | 1.018(1.008,1.028) |
|  | Lag4 | 1.002(0.994,1.010) | 1.025(1.015,1.035) | 1.014(1.006,1.023) | 1.012(1.003,1.021) |
|  | Lag5 | 0.998(0.990,1.007) | 1.017(1.007,1.028) | 1.008(0.999,1.017) | 1.007(0.998,1.017) |
|  | Lag6 | 0.995(0.986,1.004) | 1.011(0.999,1.023) | 1.003(0.993,1.012) | 1.003(0.992,1.013) |
| **CO** | Lag0 | 0.996(0.977,1.014) | 1.049(1.025,1.074) | 1.031(1.010,1.053) | 1.031(1.010,1.053) |
|  | Lag1 | 0.997(0.983,1.012) | 1.039(1.020,1.059) | 1.027(1.010,1.044) | 1.027(1.010,1.044) |
|  | Lag2 | 0.998(0.987,1.010) | 1.030(1.016,1.045) | 1.022(1.010,1.035) | 1.022(1.010,1.035) |
|  | Lag3 | 1.000(0.991,1.008) | 1.021(1.010,1.032) | 1.018(1.008,1.028) | 1.018(1.008,1.028) |
|  | Lag4 | 1.000(0.993,1.008) | 1.013(1.003,1.023) | 1.014(1.006,1.023) | 1.014(1.006,1.023) |

| **Pollutants** | **Days** | **0-5** | **6-14** | **boy** | **girl** |
| --- | --- | --- | --- | --- | --- |
| **PM_2.5_** | Lag0 | 1.012(0.993,1.030) | 1.055(1.030,1.080) | 1.021(1.002,1.042) | 1.041(1.020,1.063) |
|  | Lag1 | 1.010(0.996,1.025) | 1.044(1.024,1.063) | 1.016(1.001,1.032) | 1.035(1.019,1.052) |
|  | Lag2 | 1.009(0.998,1.020) | 1.033(1.018,1.048) | 1.011(1.000,1.023) | 1.030(1.017,1.042) |
|  | Lag3 | 1.008(1.000,1.016) | 1.023(1.012,1.034) | 1.007(0.998,1.016) | 1.024(1.015,1.034) |
|  | Lag4 | 1.007(0.999,1.014) | 1.014(1.004,1.024) | 1.003(0.995,1.011) | 1.019(1.011,1.027) |
|  | Lag5 | 1.006(0.998,1.013) | 1.006(0.997,1.017) | 1.000(0.992,1.008) | 1.014(1.006,1.023) |
| **PM_10_** | Lag0 | 1.009(0.992,1.026) | 1.035(1.012,1.058) | 1.016(0.998,1.035) | 1.025(1.006,1.044) |
|  | Lag1 | 1.008(0.995,1.021) | 1.029(1.011,1.047) | 1.013(0.999,1.027) | 1.022(1.007,1.037) |
|  | Lag2 | 1.007(0.997,1.017) | 1.023(1.010,1.036) | 1.010(0.999,1.021) | 1.019(1.008,1.031) |
|  | Lag3 | 1.005(0.998,1.013) | 1.017(1.007,1.028) | 1.007(0.999,1.015) | 1.017(1.008,1.025) |
|  | Lag4 | 1.004(0.998,1.011) | 1.013(1.004,1.021) | 1.004(0.997,1.011) | 1.014(1.006,1.021) |
|  | Lag5 | 1.003(0.997,1.010) | 1.008(0.999,1.017) | 1.002(0.995,1.010) | 1.011(1.004,1.019) |
|  | Lag6 | 1.002(0.995,1.010) | 1.005(0.995,1.015) | 1.001(0.993,1.009) | 1.009(1.000,1.017) |
| **O_3_** | Lag0 | 1.022(1.000,1.044) | 1.029(0.999,1.059) | 1.019(0.995,1.043) | 1.027(1.002,1.053) |
|  | Lag1 | 1.016(0.999,1.033) | 1.025(1.003,1.049) | 1.015(0.996,1.033) | 1.022(1.003,1.042) |
|  | Lag2 | 1.010(0.997,1.023) | 1.022(1.005,1.040) | 1.011(0.997,1.025) | 1.018(1.003,1.033) |
|  | Lag3 | 1.005(0.995,1.015) | 1.019(1.006,1.033) | 1.007(0.996,1.018) | 1.014(1.002,1.025) |
|  | Lag4 | 1.001(0.992,1.010) | 1.017(1.005,1.029) | 1.004(0.995,1.014) | 1.010(1.000,1.020) |
| **NO_2_** | Lag0 | 1.008(0.990,1.026) | 1.071(1.045,1.097) | 1.035(1.015,1.056) | 1.030(1.009,1.051) |
|  | Lag1 | 1.007(0.993,1.021) | 1.057(1.038,1.077) | 1.029(1.013,1.045) | 1.025(1.009,1.042) |
|  | Lag2 | 1.006(0.995,1.017) | 1.045(1.030,1.060) | 1.023(1.011,1.035) | 1.021(1.008,1.033) |
|  | Lag3 | 1.005(0.997,1.013) | 1.032(1.021,1.044) | 1.017(1.008,1.026) | 1.016(1.007,1.026) |
|  | Lag4 | 1.004(0.997,1.011) | 1.022(1.012,1.031) | 1.011(1.004,1.019) | 1.012(1.004,1.020) |
|  | Lag5 | 1.003(0.996,1.010) | 1.012(1.002,1.022) | 1.007(0.999,1.014) | 1.008(1.000,1.016) |
|  | Lag6 | 1.001(0.994,1.009) | 1.004(0.994,1.014) | 1.002(0.994,1.011) | 1.004(0.995,1.013) |
| **SO_2_** | Lag0 | 1.015(0.993,1.038) | 1.071(1.041,1.101) | 1.047(1.022,1.071) | 1.037(1.012,1.064) |
|  | Lag1 | 1.011(0.994,1.029) | 1.059(1.036,1.082) | 1.037(1.019,1.056) | 1.031(1.011,1.051) |
|  | Lag2 | 1.007(0.994,1.021) | 1.047(1.030,1.064) | 1.029(1.015,1.043) | 1.024(1.010,1.039) |
|  | Lag3 | 1.004(0.994,1.014) | 1.036(1.023,1.049) | 1.020(1.010,1.031) | 1.018(1.007,1.030) |
|  | Lag4 | 1.000(0.991,1.009) | 1.026(1.014,1.037) | 1.012(1.003,1.022) | 1.013(1.003,1.023) |
|  | Lag5 | 0.997(0.988,1.006) | 1.017(1.005,1.029) | 1.006(0.996,1.016) | 1.008(0.997,1.018) |
| **CO** | Lag0 | 1.001(0.980,1.022) | 1.049(1.021,1.077) | 1.019(0.997,1.042) | 1.028(1.004,1.052) |
|  | Lag1 | 1.002(0.985,1.018) | 1.039(1.018,1.061) | 1.015(0.997,1.033) | 1.024(1.006,1.043) |
|  | Lag2 | 1.002(0.990,1.015) | 1.031(1.015,1.047) | 1.011(0.998,1.024) | 1.021(1.007,1.035) |
|  | Lag3 | 1.002(0.993,1.012) | 1.022(1.010,1.035) | 1.007(0.997,1.017) | 1.017(1.006,1.028) |
|  | Lag4 | 1.002(0.994,1.011) | 1.015(1.004,1.026) | 1.003(0.994,1.013) | 1.014(1.004,1.023) |
|  | Lag5 | 1.002(0.993,1.011) | 1.008(0.997,1.019) | 1.000(0.991,1.009) | 1.010(1.000,1.020) |

Table S2. The result of subgroup analyses in children infectious respiratory disease.

Table S3. The result of subgroup analyses in children chronic respiratory disease.

| **Pollutants** | **Days** | **0-5** | **6-14** | **boy** | **girl** |
| --- | --- | --- | --- | --- | --- |
| **PM_2.5_** | Lag0 | 1.017(0.985,1.050) | 1.065(1.025,1.106) | 1.023(0.990,1.057) | 1.056(1.018,1.095) |
|  | Lag1 | 1.013(0.988,1.038) | 1.050(1.020,1.082) | 1.018(0.992,1.045) | 1.042(1.013,1.072) |
|  | Lag2 | 1.009(0.990,1.028) | 1.037(1.014,1.060) | 1.014(0.994,1.034) | 1.029(1.007,1.052) |
|  | Lag3 | 1.005(0.990,1.019) | 1.024(1.006,1.042) | 1.009(0.994,1.025) | 1.017(1.001,1.034) |
|  | Lag4 | 1.001(0.989,1.014) | 1.012(0.997,1.028) | 1.005(0.992,1.018) | 1.007(0.992,1.022) |
| **PM_10_** | Lag0 | 1.022(0.993,1.052) | 1.063(1.026,1.101) | 1.033(1.002,1.064) | 1.049(1.014,1.085) |
|  | Lag1 | 1.018(0.995,1.041) | 1.051(1.023,1.080) | 1.027(1.003,1.052) | 1.039(1.012,1.067) |
|  | Lag2 | 1.013(0.996,1.031) | 1.040(1.019,1.062) | 1.021(1.003,1.040) | 1.030(1.009,1.050) |
|  | Lag3 | 1.009(0.996,1.023) | 1.030(1.014,1.047) | 1.016(1.002,1.030) | 1.021(1.005,1.036) |
|  | Lag4 | 1.006(0.994,1.017) | 1.021(1.007,1.035) | 1.011(0.999,1.023) | 1.013(1.000,1.026) |
|  | Lag5 | 1.002(0.990,1.014) | 1.013(0.999,1.027) | 1.007(0.994,1.019) | 1.006(0.993,1.020) |
| **O_3_** | Lag0 | 1.023(0.985,1.062) | 1.037(0.991,1.084) | 1.041(1.001,1.082) | 1.018(0.975,1.063) |
|  | Lag1 | 1.017(0.987,1.047) | 1.029(0.994,1.066) | 1.033(1.001,1.065) | 1.012(0.979,1.047) |
| **NO_2_** | Lag0 | 1.020(0.988,1.053) | 1.089(1.048,1.131) | 1.042(1.008,1.077) | 1.051(1.014,1.091) |
|  | Lag1 | 1.014(0.990,1.040) | 1.071(1.039,1.104) | 1.031(1.005,1.058) | 1.042(1.012,1.072) |
|  | Lag2 | 1.009(0.990,1.028) | 1.054(1.030,1.079) | 1.021(1.001,1.041) | 1.033(1.010,1.055) |
|  | Lag3 | 1.003(0.989,1.018) | 1.039(1.021,1.057) | 1.011(0.996,1.026) | 1.024(1.007,1.041) |
|  | Lag4 | 0.999(0.987,1.011) | 1.025(1.009,1.040) | 1.003(0.990,1.016) | 1.016(1.002,1.031) |
| **SO_2_** | Lag0 | 1.033(0.989,1.079) | 1.037(0.990,1.086) | 1.041(0.997,1.086) | 1.031(0.982,1.082) |
|  | Lag1 | 1.027(0.993,1.063) | 1.032(0.996,1.070) | 1.035(1.002,1.070) | 1.024(0.986,1.063) |
|  | Lag2 | 1.021(0.995,1.048) | 1.027(1.000,1.055) | 1.030(1.005,1.056) | 1.017(0.989,1.046) |
|  | Lag3 | 1.016(0.995,1.036) | 1.023(1.002,1.044) | 1.025(1.006,1.045) | 1.011(0.989,1.033) |
|  | Lag4 | 1.010(0.993,1.028) | 1.018(1.000,1.037) | 1.020(1.003,1.038) | 1.006(0.986,1.025) |
|  | Lag5 | 1.006(0.987,1.025) | 1.014(0.995,1.034) | 1.016(0.998,1.034) | 1.001(0.981,1.022) |
| **CO** | Lag0 | 0.971(0.934,1.009) | 1.047(1.002,1.095) | 0.982(0.945,1.021) | 1.03(0.986,1.076) |
|  | Lag1 | 0.978(0.949,1.008) | 1.035(0.999,1.071) | 0.983(0.954,1.014) | 1.025(0.990,1.060) |
|  | Lag2 | 0.985(0.962,1.008) | 1.022(0.996,1.050) | 0.985(0.962,1.008) | 1.020(0.994,1.047) |
|  | Lag3 | 0.991(0.973,1.009) | 1.011(0.991,1.032) | 0.986(0.968,1.004) | 1.015(0.995,1.036) |
|  | Lag4 | 0.996(0.980,1.012) | 1.001(0.984,1.019) | 0.987(0.971,1.002) | 1.011(0.993,1.029) |
|  | Lag5 | 1.001(0.984,1.017) | 0.993(0.975,1.011) | 0.988(0.972,1.004) | 1.007(0.989,1.026) |
|  | Lag6 | 1.003(0.986,1.021) | 0.987(0.968,1.006) | 0.989(0.972,1.006) | 1.003(0.984,1.023) |

Table S4. Sensitivity analyses results of dual pollution model.

| **Pollutants** | **RD** | **IRD** | **CRD** |
| --- | --- | --- | --- |
| **PM_2.5_** | 1.032(1.018-1.047) | 1.031(1.014-1.047) | 1.036(1.010-1.063) |
| **+NO_2_** | 1.022(1.007-1.037) | 1.021(1.004-1.038) | 1.025(0.999-1.053) |
| **+SO_2_** | 1.028(1.013-1.043) | 1.026(1.010-1.043) | 1.033(1.006-1.059) |
| **+CO** | 1.024(1.008-1.040) | 1.019(1.001-1.037) | 1.042(1.013-1.071) |
| **+ O_3_** | 1.030(1.015-1.045) | 1.028(1.011-1.045) | 1.036(1.010-1.063) |
| **PM_10_** | 1.025(1.011-1.038) | 1.020(1.005-1.035) | 1.039(1.014-1.064) |
| **+ NO_2_** | 1.014(1.001-1.028) | 1.010(0.995-1.026) | 1.029(1.005-1.055) |
| **+ SO_2_** | 1.020(1.006-1.033) | 1.015(1.000-1.030) | 1.036(1.011-1.061) |
| **+CO** | 1.018(1.004-1.032) | 1.011(0.996-1.027) | 1.042(1.016-1.068) |
| **+ O_3_** | 1.023(1.010-1.037) | 1.019(1.004-1.034) | 1.039(1.015-1.064) |
| **O_3_** | 1.023(1.005-1.041) | 1.023(1.003-1.043) | 1.030(0.999-1.061) |
| **+ NO_2_** | 1.020(1.003-1.038) | 1.020(1.000-1.040) | 1.028(0.997-1.059) |
| **+ SO_2_** | 1.021(1.004-1.039) | 1.021(1.001-1.041) | 1.030(0.999-1.062) |
| **+ PM_10_** | 1.017(1.000-1.035) | 1.018(0.998-1.037) | 1.024(0.994-1.056) |
| **+CO** | 1.019(1.001-1.036) | 1.018(0.999-1.038) | 1.027(0.996-1.058) |
| **+ PM_2.5_** | 1.013(0.995-1.031) | 1.013(0.993-1.033) | 1.021(0.990-1.053) |
| **NO_2_** | 1.035(1.021-1.050) | 1.033(1.017-1.050) | 1.046(1.019-1.073) |
| **+ SO_2_** | 1.031(1.017-1.046) | 1.028(1.012-1.045) | 1.034(1.000-1.069) |
| **+ PM_10_** | 1.027(1.012-1.042) | 1.024(1.007-1.042) | 1.044(1.017-1.071) |
| **+CO** | 1.029(1.014-1.044) | 1.024(1.007-1.041) | 1.047(1.020-1.075) |
| **+ O_3_** | 1.035(1.021-1.050) | 1.033(1.016-1.049) | 1.047(1.020-1.074) |
| **+ PM_2.5_** | 1.026(1.010-1.041) | 1.022(1.005-1.040) | 1.039(1.012-1.068) |
| **SO_2_** | 1.042(1.024-1.060) | 1.042(1.022-1.062) | 1.035(1.000-1.070) |
| **+ NO_2_** | 1.031(1.013-1.050) | 1.032(1.011-1.052) | 1.025(0.991-1.060) |
| **+ PM_10_** | 1.032(1.014-1.051) | 1.033(1.012-1.053) | 1.024(0.990-1.060) |
| **+CO** | 1.035(1.017-1.053) | 1.033(1.013-1.054) | 1.036(1.001-1.071) |
| **+ O_3_** | 1.041(1.022-1.059) | 1.040(1.020-1.061) | 1.035(1.000-1.070) |
| **+ PM_2.5_** | 1.030(1.011-1.048) | 1.030(1.009-1.051) | 1.024(0.989-1.060) |
| **CO** | 1.020(1.003-1.036) | 1.025(1.007-1.043) | 1.003(0.973-1.034) |
| **+ NO_2_** | 1.010(0.994-1.027) | 1.014(0.996-1.033) | 0.990(0.960-1.021) |
| **+ SO_2_** | 1.016(0.999-1.032) | 1.019(1.001-1.038) | 0.998(0.968-1.029) |
| **+ PM_10_** | 1.009(0.992-1.026) | 1.012(0.993-1.031) | 0.993(0.962-1.025) |
| **+ O_3_** | 1.018(1.001-1.034) | 1.021(1.002-1.039) | 1.002(0.972-1.033) |
| **+ PM_2.5_** | 1.004(0.986-1.021) | 1.006(0.987-1.026) | 0.989(0.957-1.022) |

Table S5. Sensitivity analyses results of changing degree of freedom.

| **Pollutants** | **df** | **RD** | **IRD** | **CRD** |
| --- | --- | --- | --- | --- |
| **PM_2.5_** | 6 | 1.025(1.009-1.041) | 1.023(1.005-1.041) | 1.033(1.007-1.059) |
|  | 7 | 1.026(1.010-1.041) | 1.023(1.005-1.041) | 1.035(1.009-1.062) |
|  | 8 | 1.032(1.018-1.047) | 1.031(1.014-1.047) | 1.036(1.010-1.063) |
|  | 9 | 1.027(1.012-1.041) | 1.023(1.007-1.039) | 1.039(1.013-1.067) |
|  | 10 | 1.021(1.007-1.035) | 1.016(1.001-1.032) | 1.035(1.009-1.063) |
| **PM_10_** | 6 | 1.030(1.016-1.045) | 1.027(1.011-1.043) | 1.043(1.018-1.067) |
|  | 7 | 1.025(1.012-1.040) | 1.022(1.006-1.038) | 1.039(1.015-1.064) |
|  | 8 | 1.025(1.011-1.038) | 1.020(1.006-1.036) | 1.039(1.015-1.064) |
|  | 9 | 1.022(1.009-1.035) | 1.016(1.002-1.031) | 1.043(1.019-1.068) |
|  | 10 | 1.020(1.007-1.033) | 1.014(0.999-1.028) | 1.044(1.019-1.069) |
| **O_3_** | 6 | 1.018(1.000-1.037) | 1.017(0.996-1.039) | 1.030(0.999-1.062) |
|  | 7 | 1.016(0.997-1.034) | 1.014(0.994-1.036) | 1.026(0.996-1.058) |
|  | 8 | 1.023(1.005-1.041) | 1.023(1.003-1.043) | 1.030(0.999-1.061) |
|  | 9 | 1.024(1.007-1.041) | 1.025(1.006-1.045) | 1.024(0.993-1.056) |
|  | 10 | 1.027(1.010-1.044) | 1.030(1.011-1.049) | 1.023(0.992-1.054) |
| **NO_2_** | 6 | 1.051(1.036-1.067) | 1.048(1.030-1.066) | 1.066(1.040-1.093) |
|  | 7 | 1.045(1.030-1.061) | 1.043(1.026-1.061) | 1.055(1.028-1.082) |
|  | 8 | 1.035(1.021-1.050) | 1.033(1.017-1.050) | 1.046(1.019-1.073) |
|  | 9 | 1.033(1.019-1.048) | 1.029(1.013-1.046) | 1.050(1.023-1.077) |
|  | 10 | 1.035(1.021-1.050) | 1.031(1.015-1.047) | 1.054(1.026-1.081) |
| **SO_2_** | 6 | 1.046(1.027-1.065) | 1.046(1.025-1.068) | 1.044(1.011-1.079) |
|  | 7 | 1.053(1.034-1.072) | 1.053(1.031-1.074) | 1.048(1.013-1.083) |
|  | 8 | 1.042(1.024-1.060) | 1.042(1.022-1.063) | 1.035(1.001-1.070) |
|  | 9 | 1.034(1.016-1.052) | 1.030(1.011-1.050) | 1.041(1.007-1.077) |
|  | 10 | 1.021(1.003-1.038) | 1.015(0.996-1.035) | 1.040(1.005-1.075) |
| **CO** | 6 | 1.018(1.000-1.036) | 1.023(1.003-1.043) | 0.996(0.966-1.026) |
|  | 7 | 1.015(0.998-1.033) | 1.018(0.999-1.038) | 1.001(0.970-1.032) |
|  | 8 | 1.020(1.003-1.036) | 1.023(1.005-1.042) | 1.003(0.973-1.034) |
|  | 9 | 1.022(1.006-1.039) | 1.025(1.007-1.043) | 1.009(0.979-1.040) |
|  | 10 | 1.018(1.002-1.034) | 1.021(1.003-1.039) | 1.003(0.972-1.034) |
